# Complete Genome Sequence of Bovine Viral Diarrhea Virus Subgenotype 2a Strain CN10.2015.821, Isolated in Piedmont, Italy

**DOI:** 10.1128/genomeA.00170-18

**Published:** 2018-03-15

**Authors:** B. Colitti, C. Nogarol, L. Bertolotti, S. Rosati

**Affiliations:** a Department of Veterinary Science, University of Turin, Grugliasco, Italy

## Abstract

We sequenced the complete genome of noncytopathic bovine viral diarrhea virus (BVDV) type 2 strain CN10.2015.821. It belongs to the subgenotype 2a, and it was isolated from an immunotolerant and persistently infected calf identified during a serological investigation. The complete genome is composed of 12,273 nucleotides, organized as one open reading frame encoding 3,897 amino acids.

## GENOME ANNOUNCEMENT

Bovine viral diarrhea virus (BVDV) is an important pathogen of ruminants (1), causing severe economic losses to the cattle industry (2). The severity of clinical signs is strictly associated with the viral strain and the type of infection. In most cattle transiently infected with BVDV, disease signs are mild, characterized by low-grade fever, diarrhea, and coughing (3, 4).

BVDV types 1 and 2 belong to the genus Pestivirus of the family *Flaviviridae*, along with border disease virus (BDV) and classical swine disease virus (4). Other atypical pestiviruses include HoBi-like virus, which is tentatively classified as BVDV type 3 (5, 6), and wild ungulate pestiviruses.

In the current study, we determined the full-length genome sequence of noncytopathic strain BVDV-2a CN10.2015.821. The isolate was obtained from a persistently infected (PI) calf identified during a serological investigation in the Piedmont region of Italy in 2014 (7).

Animal blood serum was used to infect pestivirus-free Madin-Darby bovine kidney epithelial cell culture. The supernatant of the second culture passage was collected and used for viral RNA extraction (QIAamp Viral RNA minikit; Qiagen, Hilden, Germany). Double-stranded cDNA was obtained (Maxima H Minus double-stranded cDNA synthesis kit; Thermo Fisher Scientific), and libraries were prepared using the Illumina Nextera XT protocol (Illumina, San Diego, USA). The prepared libraries were evaluated with the Bioanalyzer 2100 high sensitivity chip (Agilent). Paired-end sequencing was performed using the Illumina MiSeq platform. The reads generated were analyzed using an “assembling *de novo*” approach (Velvet version 1.2.10) and by resequencing, using available full genome sequences as references (Geneious version 11.0.2).

The complete genome of the strain CN10.2015.821 comprises 12,273 nucleotides (nt), with 5′ and 3′ untranslated regions (UTRs) of 386 nt and 193 nt, respectively. The single large open reading frame encodes 3,897 amino acids.

Compared to reference sequences, the virus shares from 84.4% (GenBank accession no. KJ000672) to 95.2% (GenBank accession no. JN380086) nucleotide similarity with other published full-length BVDV-2 genome sequences. The large open reading frame encodes four structural proteins (C [nt 891 to 1196], E^rns^ [nt 1197 to 1877], E1 [1878 to 2462], and E2 [nt 2463 to 3578]) and seven nonstructural proteins (N^pro^ [nt 387 to 890], p7 [nt 3579 to 3788], NS2/3 [nt 3789 to 7196], NS4A [nt 7197 to 7388], NS4B [nt 7389 to 8429], NS5A [nt 8430 to 9920], and NS5B [nt 9921 to 12077]).

The publication of a new full-length BVDV-2 sequence will improve knowledge in diagnostics and disease control, giving valuable molecular data to trace the source of infection and mode of transmission and to aid in vaccine selection.

### Accession number(s).

The genomic sequence of BVDV CN10.2015.821 has been deposited in GenBank under the accession no. MG879027.
